# Characteristics of an Implantable Blood Pressure Sensor Packaged by Ultrafast Laser Microwelding

**DOI:** 10.3390/s19081801

**Published:** 2019-04-15

**Authors:** Sungil Kim, Jaesoon Park, Sangkyun So, Sanghoon Ahn, Jiyeon Choi, Chiwan Koo, Yeun-Ho Joung

**Affiliations:** 1 Department of Electronics and Control Engineering, Hanbat National University, Daejeon 34158, Korea; sung1@hanbat.ac.kr (S.K.); wotns3645@naver.com (J.P.); sanggso@hanbat.ac.kr (S.S.); 2 Department of Laser and Electron Beam Application, Korea Institute of Machinery and Materials, Daejeon 34103, Korea; shahn@kimm.re.kr

**Keywords:** implantable blood pressure sensor, ultrafast laser, glass welding, direct bonding, MEMS hermetic packaging

## Abstract

We propose a new packaging process for an implantable blood pressure sensor using ultrafast laser micro-welding. The sensor is a membrane type, passive device that uses the change in the capacitance caused by the membrane deformation due to applied pressure. Components of the sensor such as inductors and capacitors were fabricated on two glass (quartz) wafers and the two wafers were bonded into a single package. Conventional bonding methods such as adhesive bonding, thermal bonding, and anodic bonding require considerable effort and cost. Therefore CO_2_ laser cutting was used due to its fast and easy operation providing melting and bonding of the interface at the same time. However, a severe heat process leading to a large temperature gradient by rapid heating and quenching at the interface causes microcracks in brittle glass and results in low durability and production yield. In this paper, we introduce an ultrafast laser process for glass bonding because it can optimize the heat accumulation inside the glass by a short pulse width within a few picoseconds and a high pulse repetition rate. As a result, the ultrafast laser welding provides microscale bonding for glass pressure sensor packaging. The packaging process was performed with a minimized welding seam width of 100 μm with a minute. The minimized welding seam allows a drastic reduction of the sensor size, which is a significant benefit for implantable sensors. The fabricated pressure sensor was operated with resonance frequencies corresponding to applied pressures and there was no air leakage through the welded interface. In addition, in vitro cytotoxicity tests with the sensor showed that there was no elution of inner components and the ultrafast laser packaged sensor is non-toxic. The ultrafast laser welding provides a fast and robust glass chip packaging, which has advantages in hermeticity, bio-compatibility, and cost-effectiveness in the manufacturing of compact implantable sensors.

## 1. Introduction

Hypertension is a major risk factor for cardiovascular disease (CVD) and stroke, causing disability and premature death worldwide. Over the last 30 years, ongoing clinical treatments and control of hypertension have been conducted, but they have failed to reduce the proportion of adults with hypertension [1]. In order to resolve this issue, it is necessary to reduce the misdiagnosis by removing the erroneous factors providing incorrect measurements such as patient posture, environment, cuff size, and measurement technique and by measuring blood pressure accurately before and after the treatment [2,3]. Even though standardized non-invasive cuff and catheterized invasive devices are utilized in favorable conditions, misinterpretation of the blood pressure has occurred due to white coat effects produced by complications such as the patients’ lack of comfort, trauma, and infection [4,5,6].

The development of a variety of biological signal measurement technologies using wearable or implantable biosensors promises to change the conventional medical treatment based on a single measurement into prophylactic treatment through real-time continuous monitoring with high accuracy and precision [7,8,9]. An implantable blood pressure sensor is inserted into blood vessels to directly monitor the blood vessel pressure over a long period of time, and the long-term data can be used for the evaluation of hypertension, heart failure, restenosis, and the efficacy of surgical interventions [4,10]. Over the past decades, implantable blood pressure sensors have been developed by various groups focusing on principles, materials, and fabrication processes [10,11,12,13,14,15,16,17,18,19]. Most blood pressure sensors use a micro-scale membrane that is deformed under external pressure. The membrane works as a capacitor and the capacitance varies with physical pressure and is measured as an electrical signal. The advantages of this capacitive structure are a simple structure and easy integration with other components [20,21]. However, the implantable sensor requires a perfectly hermetic package because the electronic components of the sensor should be completely isolated from the living body. In addition, the biocompatibility of packaging materials also should be considered.

We have developed an implantable blood pressure sensor that satisfies all of the above conditions with biocompatibility. Moreover, the sensor size was minimized without a battery to reduce possible harmful factors. Quartz glass (SiO_2_) was chosen for the upper and bottom substrates of the sensor because it exhibits excellent material properties in terms of mechanical, chemical, thermal, optical, and insulation characteristics. In particular, it has high biocompatibility and thus is frequently used for implantable medical devices and bio-MEMS devices [22,23,24]. However, glass is in general brittle and consequently has many limitations related to micromachining such as micro-patterning using MEMS processes, hole-drilling using diamond drill bits, and mechanical cutting using blades. In particular, a lack of reliable bonding technology limits glass-based device development [24,25,26]. Typically, simple and easy glass-to-glass bonding methods with an adhesive layer such as epoxy and UV glue are used in most pressure sensor fabrications. However, encapsulation of implantable sensors with chemical adhesives is not suitable due to low mechanical strength, thermal resistance, and chemical durability of the intermediate adhesive layer [27]. On the other hand, direct bonding methods such as thermal bonding, fusion bonding, and anodic bonding are hermetic and provide high bonding strength. However, they do not provide selective bonding of the specific area and require long processing time and are costly. In particular, thermal bonding requires a high temperature (~550 °C) and fusion bonding uses both high pressure and high voltage. Therefore, direct bonding or adhesive bonding is not capable of packaging sensors with built-in components such as metal inductors [28].

In the case of initial implantable blood pressure sensors, CO_2_ laser packaging was applied to overcome the limitations of conventional packaging processes [10,11,12,18]. The advantage of adopting the CO_2_ laser process is that the glass is very absorptive at the wavelength of the laser and thus the temperature at the cutting edge is rapidly increased over the melting point of the glass during laser cutting to separate individual sensors from a wafer. The local heating of edges followed by rapid quenching after melting of the top and bottom substrates leads to bonding of the interface [29]. However, the use of brittle materials such as glass, excessive heat accumulation, and propagation during the process cause microcrack formation at the edge, which significantly reduces the yield and durability of the sensors. Therefore, generation of a large heat-affected zone (HAZ) around the laser focus is a bottleneck to the miniaturization of sensors [30].

Recently, ultrafast lasers have been widely used in the fabrication of glass-based microdevices. Ultrafast laser micromachining provides advantages that are particularly useful for glass micromachining. First, ultrafast lasers including femtosecond lasers deliver photon energy to glass substrates very efficiently within their pulse duration, which is much shorter than the heat transfer time through a glass. Thus, the process minimizes the HAZ around the process area [31]. Second, nonlinear absorption induced by the ultrafast laser enables effective absorption of photon energy in the vicinity of the focused beam in the glass. The localized absorption leads to local melting of glass only at the interface between glass substrates. This allows direct bonding of glass without chemical adhesives or physical processes such as heating of glass with pressure [32,33,34,35]. In 2005, direct welding of glass was first demonstrated by Tamaki et al. with silica glass using a Ti:sapphire femtosecond laser at a repetition rate of 1 kHz and a pulse width of 85 fs [32]. However, due to the limitation of the process speed by the low pulse repetition rate and its poor bonding strength, direct bonding using ultrafast lasers has not been practically applied. Recently, process optimization has been achieved to overcome the limitations by effective control of heat accumulation with the introduction of a high pulse repetition rate laser system in the MHz range. The direct bonding of glass using ultrafast lasers has opened a new door to innovate the fabrication of glass-based devices [24,26,35,36,37].

The advantages of ultrafast laser welding for implantable blood pressure sensor packaging are as follows: First, the sensor size can be drastically reduced as welding seams can be placed as close as possible to the sensing areas on a microscale [38]. Second, the durability of the device can be improved thanks to high bonding strength [39]. Our preliminary research demonstrates that the obtained maximum internal pressure of a glass microfluidic device was as high as 1.4 MPa and leak-free packaging was also realized [24]. Other prior studies have reported that the maximum bonding strength was roughly 85% of the pristine bulk material [40]. Lastly, simple and rapid direct bonding increases the process efficiency and improves biocompatibility as no chemical interlayer is required [41,42]. 

In this paper, direct welding of glass using an ultrafast laser at a high pulse repetition rate is applied to improve the packaging process of an implantable blood pressure sensor. We investigated optimal welding conditions in terms of critical laser processing parameters such as beam scanning speed, pulse energy, and focal position. We then analyzed the resonant frequency characteristics of the fabricated sensor corresponding to the precision pressure control range of blood pressure. As a result, fast and reliable packaging of glass implantable sensors by ultrafast laser welding can be carried out within a minute with minimal welding seam on a microscale.

## 2. Experiments

### 2.1. Principle and Fabrication of Implantable Blood Pressure Sensor

A real-time implantable blood pressure sensor requires biocompatibility of the sensor material and ease of wireless communication. Therefore, we selected an LC resonant-type pressure sensor on a quartz wafer (Semistore, Pyeongtaek, Korea) that is inductively coupled with an external antenna coil of a measurement system and this passive type sensor does not require a battery [43]. 

**Figure 1:**
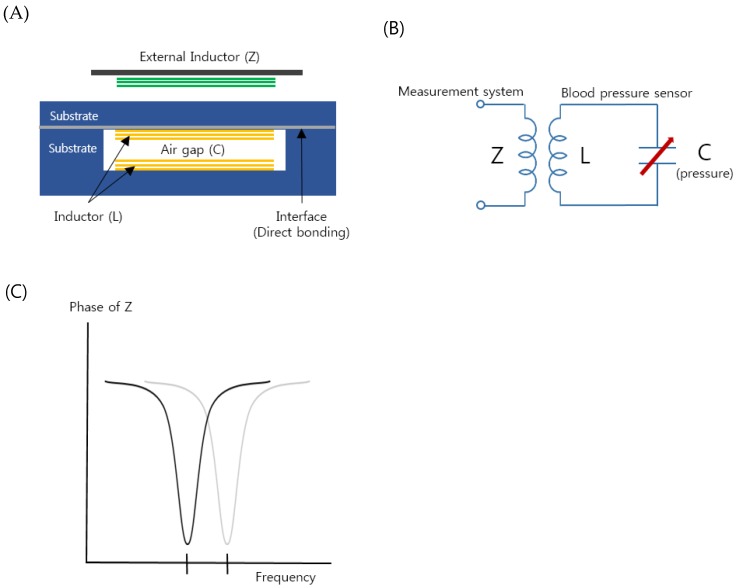
(**A**) Schematic of the proposed implantable blood pressure sensor with two micro-inductors with an air gap working as a capacitor. (**B**) Equivalent circuit of the implantable blood pressure sensor. (**C**) Variation of the sensor resonance frequency by increasing pressure (in vascular).

Figure 1A shows a schematic illustration of the proposed implantable blood pressure sensor consisting of two inductor coils. They are placed such that they face each other with an air gap and this configuration works as a capacitor so that the entire structure is equivalent to an LC resonance circuit, as shown in Figure 1B. When an external pressure is applied to the hermetic sensor package, one thin wall of the package is deformed and the air gap is also changed. The change varies the capacitance of the resonance circuit, resulting in a resonant frequency shift [44]. To monitor the altered resonant frequency, an external inductor, which is placed near the sensor package, indirectly measures the impedance of the resonance circuit as a function of frequency (Figure 1C). As the external inductor and the pressure sensor are inductively coupled, the frequency when the maximum energy is transferred from the external inductor to the pressure sensor is the resonant frequency of the pressure sensor. If the frequency is measured, the change in the capacitance of the resonance circuit is calculated and the external pressure applied to the sensor is measured. It is not necessary to have an active power source such as batteries in the presented pressure sensor so that the sensor is a passive type, as opposed to active sensors that have their own power source.

The two micro inductors were fabricated on two quartz substrates; one is a flat thin substrate and the other has a trench (Figure 1A). A quartz material was selected due to its high chemical resistance and non-toxicity. The micro-inductor fabrication was conducted using MEMS processes such as sputtering, etching, photolithography, electroplating, bonding, and so on [10,11,12,18,19]. The detailed fabrication steps are described in Supplementary Material. The micro-inductor has 10 turns of a wire with a diameter of 60 μm and a spacing of 40 μm. The size of the fabricated inductors was 2.2 mm (width) × 12 mm (length). The glass substrates were directly bonded by optical contact [19]. 

### 2.2. Optimization of Micro Welding for Implantable Blood Pressure Sensor Packaging

**Figure 2:**
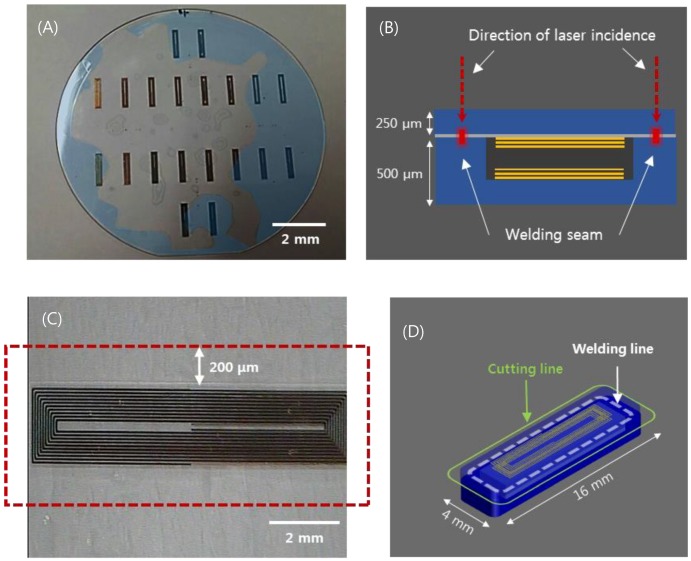
(**A**) Wafer-state of sensors fabricated by MEMS process. (**B**) depiction of the laser welding (sensor thickness 750 μm). (**C**) visualization of the welded area near the inductor of a sensor. (**D**) visualization of the laser cut path for individualization and miniaturization of sensors (sensor cutting size: width 4mm, length 16 mm).

The aforementioned implantable blood pressure sensor was pre-bonded (optical contact), as shown in Figure 2A, to ensure hermetic sealing. The glass can be bonded directly by van der Waals force without adhesive if the cleanliness of the bonding surface is maintained throughout chemical cleaning [45]. However, an irregularly bonded area (interference fringes, indicated by blue area) is observed due to fine gaps generated by metal and organic particles or dust, which are residues caused by imperfect cleaning. This is the primary factor that degrades the sealing quality of the sensor. This degradation of the sealing quality can be mitigated by using ultrafast laser welding. Prior studies showed that ultrafast laser glass welding can effectively bond glass substrates by filling the interface gap up to 3 μm [46,47]. Figure 2B provides a schematic illustration of the ultrafast laser welding of a cover glass to the sensor substrate. One side of the substrate (membrane) changes the capacitance according to the mechanical deformation by pressure. Therefore, thickness control of the upper wafer was performed to improve the sensitivity. We first investigated the feasibility of glass wafer welding with an optical contacted area and non-optical contacted area. The laser used for this experiment was a femtosecond laser (Satsuma HP2, Amplitude Systèmes, Pessac, France) with a center wavelength at 1030 nm.

This laser provides an adjustable pulse width between 0.3 to 10 picoseconds (ps) and the pulse repetition rate is variable up to 2 MHz. In most cases, the pulse width was set to be 1 ps at a repetition rate of 2 MHz to introduce heat accumulation. Modest heat accumulation can effectively melt the glass at the vicinity of the laser focus between substrate interfaces. The laser process parameters such as pulse energy, focal position, and beam scanning speed have been quantitatively investigated to optimize welding conditions. Pulse energy ranging from 2 to 8 μJ and scanning speed from 10 to 40 mm/s were tested to determine the process window. A 3D machining stage with a maximum translation speed of 300 mm/s and 300 mm full stroke was used to mount and translate the glass wafer. The flatness of the 4-inch wafer was kept within 5 μm. The focused laser beam was initially placed 450 μm below the interface and moved towards the interface by 50 μm after a scan to explore the effect of the focus position on the welding quality. A 20× objective lens (378-867-5, Mitutoyo, Kawasaki, Japan) with a numerical aperture of 0.4 was used to focus the laser beam. Figure 2C shows the welded zone of selective welding near the micro-inductor components of the sensor. After laser welding, laser cutting was performed by the same laser source to separate the sensors from the glass wafer. However, the pulse width was adjusted to be the shortest, i.e. about 370 fs, to minimize the thermal effect. Figure 2D illustrates the welding and cutting paths. Finally, we compared the quality of the cross sections of two sensors, which were separated using either a CO_2_ laser or an ultrafast laser. 

### 2.3. Evaluation of Improved Packaging 

**Figure 3:**
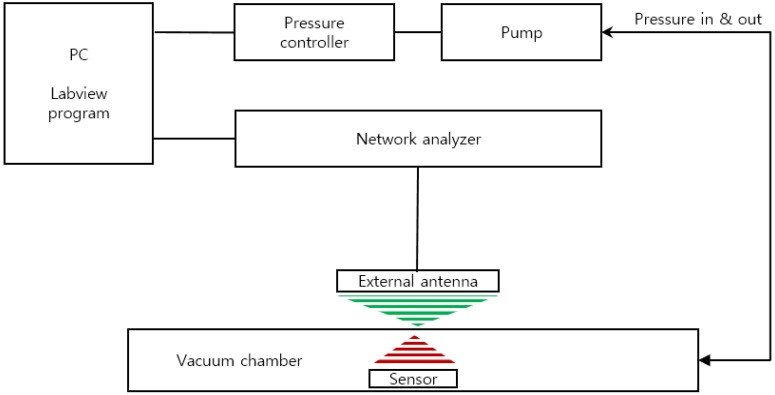
Schematic of the sensor characterization evaluation system.

We developed a custom-built performance evaluation system for the measurement of the resonant frequency of the sensor according to the change in pressure (Figure 3). It is composed of an ultra-precision pressure controller (CPC3000, Mensor, San Marcos, TX, USA), vacuum and pressure pumps, a custom-built chamber, and a network analyzer (8753E, Hewlett Packard (HP), Palo Alto, CA, USA). The entire device was controlled by LabVIEW programming and the data of pressure and frequency were through GPIB communication (IEEE-488). The input and output characteristics of the implantable blood pressure sensor at different pressures were analyzed by the customized evaluation system. For the pressure protocol of the input value, the pressure range from 760 mmHg to 900 mmHg was selected considering the human blood pressure range and the atmospheric pressure difference. While the input pressure was increased to 15 steps by 10 ± 1 mmHg and then decreased by the same interval, the resonance frequency was measured at each step. At each pressure step, the sensor was exposed to a dwelling time of 2 min for mechanical stabilization before measuring the resonance frequency.

The resonant frequencies of the non-welded and non-cut sensor were measured first, and then the sensor was welded and cut and the resonant frequencies were measured. With the resonance frequency difference, we analyzed the input-output characterization, sensitivity, and hysteresis.

We assessed the in vitro cytotoxicity of the sensor according to ISO 10993-5: 2009. The biocompatibility of SiO_2_ material has already been well established in many studies [48]. If there are any physical defects in the sensor packaging, the eluate from the internal components of the sensor may lead to severe human injury. In this study, we conducted an extraction method to check the biocompatibility of the packaged sensor system. The mammalian mouse fibroblast cell line, L929 (ATCC CCL1, NCTC Clone 929), was used because it can be easily cultured in a reproducible manner, and also this cell line is widely used for preliminary cytotoxicity evaluation for a wide range of biomaterials. The L929 fibroblast cells were cultured with RPMI Medium (Steinheim, Germany) and 10% Fetal Bovine Serum (FBS; GIBCO BRL, Grand Island, NY, USA) and incubated at 37 °C in 5% CO_2_ and a humid environment. The collected L-929 cells were then plated in 24-well microculture plates at a density of 1 × 10^5^ cells/ml. Extracts were obtained by placing the negative control group (high-density polyethylene film, HDPE), the positive control group (polyurethane film, ZDBC), and the test group (the fabricated sensor) in the culture media, RPMI with 10% FBS for 24 h at 37 °C. Then, the extracts were added in the microculture plates to determine their viability. After 24 h, the cells were observed for visible signs of toxicity and analyzed quantitatively using optical density.

## 3. Results and Discussion

### 3.1. Optimized Laser Conditions for Quartz Wafer Welding 

**Figure 4:**
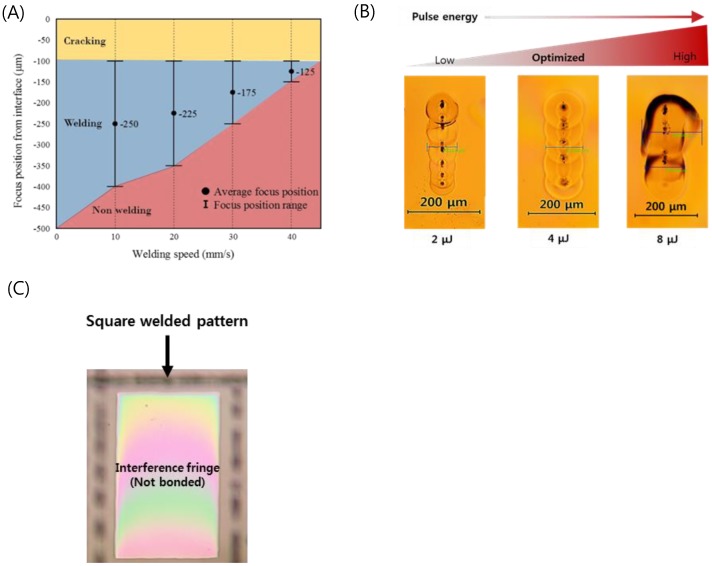
(**A**) Weldability map as functions of welding speed and focus position (E_p_ = 4 μJ). (**B**) Optical images of welding seams with different laser pulse energies (Focal position from an interface: −225 μm, Speed: 20 mm/s). (**C**) Selectively bonded square welded by optimized laser parameters (fringe-removed lines indicate successful bonding).

Welding parameter optimization of the optically contacted quartz wafer was investigated in terms of pulse energy, process speed, and focal position. Figure 4A shows the welding success range of the pulse energy of 4 μJ as a function of speed and focus position. The local glass melting by the focused ultrafast laser beam associated with volume expansion of the molten pool results in ejection of the molten pool of glass near the subsurface of glass [40]. The interface gap is filled by the ejected molten glass [40,46,47]. The molten glass volume decreases as the scanning speed increases to lower the laser dose that is related to the decrease of heat accumulation. As a result, with faster welding speed, a smaller welding seam is formed when fixed pulse energy was used. We determined that the optimal welding speed is about 20 mm/s at a focus position of 225 μm.

Figure 4B shows a top view of the welding seam with different pulse energies at the optimized processing speed and focus position. A welding seam width of 111 μm was obtained at a pulse energy of 4 μJ, which provides the best welding quality. In the case of the pulse energy of 2 μJ, the welding seam was measured to be 83 μm, which was the narrowest in this investigation. When the pulse energy was increased up to 8 μJ, excessive heat accumulation generated microcracks near the welding seam. In addition, black spots were observed in the welding seam when we used pure silica glass (e.g., quartz, fused silica), which has a higher melting point compared to other silicate glass families such as borosilicate and soda-lime glass. These spots are due to gas bubbles or disruption caused by rapid temperature rise followed by quenching at the focal volume of the glass. This may lead to degradation of the bonding strength, and therefore the process parameters should be thoroughly optimized to minimize the formation of black spots [49,50].

From these optimization processes, we found that if the glass was optically contacted and the stage flatness was kept to be smaller than 125 μm, we could form long welding seams over 100 μm, which guarantees reliable welding for entire glass substrates. Figure 4C shows a rectangularly sealed area of the quartz wafer by laser welding. The disappearance of the interference fringe (Newton’s ring) at the welded area indicates that the interfacial gap was completely filled by molten glass and thus no gap was found.

### 3.2. Implantable Blood Pressure Sensor Hermetic Packaging 

**Figure 5:**
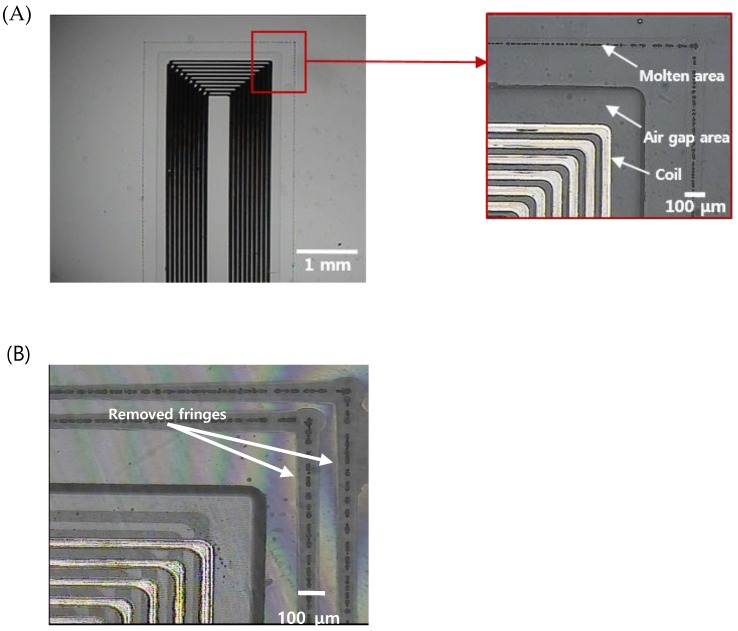
(**A**) An optical image of the sealed implantable blood pressure sensor using ultrafast laser micro-welding (the total length of the micro-welded track is 31.2 mm) (**B**) zoomed image of the corner indicating successful welding (discontinuity of interference pattern due to the welding track is shown).

Figure 5 shows the results of sealing and cutting of the sensors, which are the final steps for the fabrication of an implantable blood pressure sensor. Figure 5A shows the laser-welded sensor with good optical contact. Ultrafast laser welding can be accomplished by two approaches, continuous welding or point welding. The point welding method may be a better option for obtaining higher bonding strength [50]. However, in our case, the hermeticity of the sensor was the highest priority and thus we applied a continuous welding method to ensure a hermetically sealed sensor. Therefore, we produced a welding seam with a 100 μm width 200 μm from the air gap area where the MEMS inductor is formed. We did not observe any welding failures such as thermal damage of the MEMS inductor made of electroplated copper or physical collapse of the air gap area fabricated by wet etching.

In addition, the entire processing time required to seal a pressure sensor was within 40 s. We tried to package the sensor with a wide gap bwtween the upper and lower substrate based on the preliminary test results. Figure 5B shows that two welding seams around the inner components of the sensor were formed, and the interference fringes were selectively removed from the laser-welded regions, indicating that the bonding was successful and the interfacial gaps were completely removed. 

### 3.3. Chracterization of the Implantable Blood Pressure Sensor after Welding

**Figure 6:**
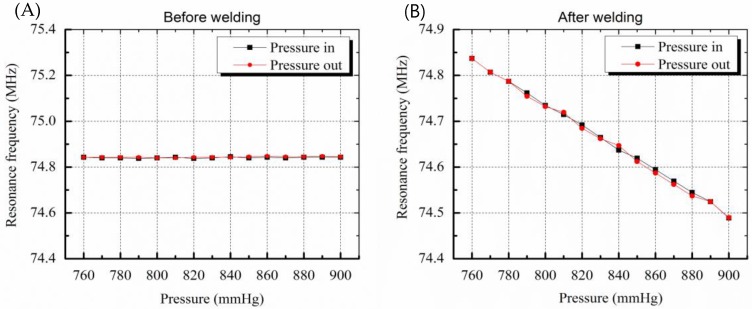
Graphs showing resonance frequency change by pressure variation. (**A**) Before welding, resonance frequency change as a function of membrane deformation caused by pressure variation is not shown due to leakage (**B**) resonance frequency variation due to deformation of membrane is clearly measured after welding.

The resonance frequency of the sensor before welding was 74.84 MHz at atmospheric pressure of 760 mmHg. As shown in Figure 6A, frequency changes of 3 kHz or less occurred randomly even though there were 15 step input pressure changes. This is due to a pressure inflow into the sensor that did not have hermetic sealing. The pressure inflow led to equilibrium of pressures between the inside and outside of the sensor and the membrane of the sensor was not deformed, resulting in no change in the capacitance of the sensor. On the other hand, the sensor after welding worked successfully and the resonance frequency linearly decreased when the pressure was increased, as in the case of the electrostatic pressure sensor. The sensitivity of the sensor was 2.4 kHz/mmHg and the error was less than 3 mmHg due to the hysteresis characteristic of 7.5 kHz. 

The hysteresis results include the performance limits of the frequency measuring system and the signal noise. The error of the sensor is lower than the error rate of the conventional cuff method, that is, 3 mmHg. As a result, the sensor pressure characteristic results show that the micro-welding provides high-quality hermetic sealing packaging. 

**Figure 7:**
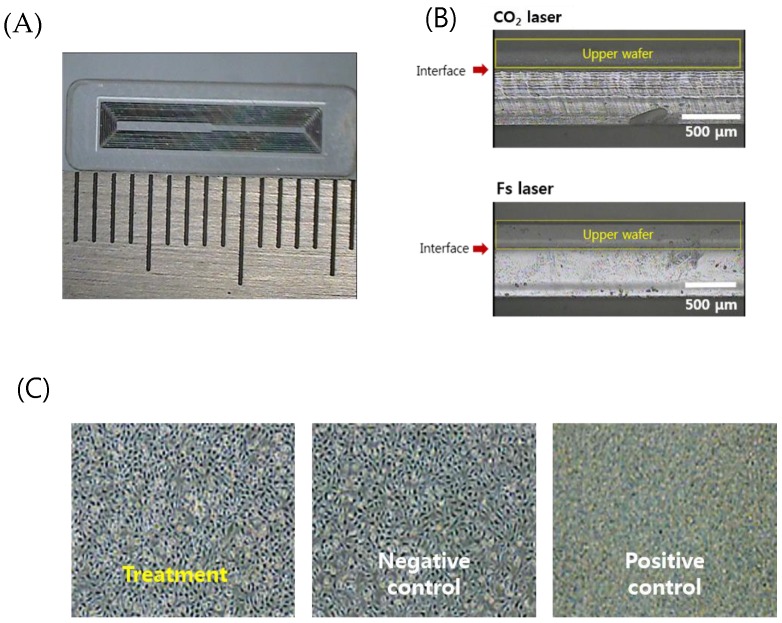
(**A**) An optical image of the separated sensor using the laser cutting (4 mm × 16 mm × 0.75 mm). (**B**) Comparison of the laser-cut cross sections by the CO_2_ laser cutting and femtosecond laser cutting. (**C**) Cytotoxicity test results for biocompatibility of welded sensors (non-toxic, grade 0).

For separation of individual sensors from the glass wafer, sensors were cut to a designed size (4 mm × 16 mm × 0.75 mm for the femoral artery), as shown in Figure 7A. The laser pulse energy for laser cutting was 30 μJ to enable high-speed machining. A Galvano scanner (IntelliSCAN III, ScanLab, Puchheim, Germany) and an f-theta lens (focal length 100 mm, Sill Optics, Wendelstein, Germany) were used for fast beam scanning. The rim of the sensor was scanned with 30 iterations at a speed of 300 mm/s. The total cutting time was about two min.

Figure 7B shows a comparison of the side view of the edge cut with a CO_2_ laser and an ultrafast laser. The upper substrate that serves as a sensor membrane was wet-etched to adjust the thickness to 250 μm so that it can be easily deformed. CO_2_ laser cutting generated irregular microcracks and thermal deformation. On the other hand, the ultrafast laser process based on ablation with 370 fs pulses has a small HAZ as well as minimized cracks and deformation. Increased cutting quality by femtosecond laser glass cutting benefits the durability of the sensor. It is well documented in fracture research that the fracture force is inversely related to the radius of the crack, and thus microcracks can be easily propagated and lead to fracture of the implanted sensor in the patient’s body. A cytotoxicity assessment to verify the safety of the implantable sensor in the body was then conducted. 

Figure 7C shows the relative cell viability (RCV) of the cells after treatment with different eluates from the negative control group, the positive control group, and the test (treatment) group, respectively, for quantitative analysis. As a result, the negative control group (high-density polyethylene film, HDPE) showed a value of 111.87% and the positive control group (ZDBC polyurethane film) showed a value of 31.55%. In the positive control group, apoptosis was clearly observed. The test group showed a RCV of 96.88% and no reactivity, no cell lysis, and no reduction of cell growth was observed (grade 0). Typically, if the cell viability is above 80–85%, the material is considered to be non-toxic, based on the ISO standard. 

## 4. Conclusions

The implantable blood pressure sensor consists of inductors and a capacitor fabricated by a MEMS process on a glass substrate and the glass substrate also served as the package structure. The biggest problem in glass micromachining using a conventional CO_2_ laser is microcracks and deformation in packaging. This generates limitations of miniaturization of implantable sensors, deterioration of airtightness, and degradation of durability. To solve this problem, we proposed a glass micro-welding strategy using an ultrafast laser and analyzed various characteristics such as hysteresis and hermeticity. The laser welding was optimized after testing with various laser pulse energy, processing speed, and focal position. As a result, the packaging by ultrafast laser micro-welding for implantable blood pressure is simpler and faster than the conventional bonding methods and provides high chemical resistance and a highly hermetic seal. The fabricated implantable blood pressure sensor works successfully with high precision and accuracy and the RCV test shows that the pressure sensor is biocompatible. Future work will evaluate the durability of the welding through an acceleration test and a helium leak test to verify long-term implantation. In addition, we expect that welding with an ultrafast laser can be applied to various glass devices such as microfluidic devices, optoelectronic devices, and optical components.

## Supplementary Materials

The following are available online at https://www.mdpi.com/1424-8220/19/8/1801/s1, Figure S1: Implantable sensor fabrication flow.

## References

[1] Carey R.M., Calhoun D.A., Bakris G.L., Brook R.D., Daugherty S.L., Dennison-Himmelfarb C.R., Lackland D.T. (2018). Resistant hypertension: Detection, evaluation, and management: A scientific statement from the American Heart Association. Hypertension.

[2] Whelton P.K., Carey R.M., Aronow W.S., Casey D.E., Collins K.J., Himmelfarb C.D., MacLaughlin E.J. (2018). ACC/AHA/AAPA/ABC/ACPM/AGS/APhA/ASH/ASPC/NMA/PCNA guideline for the prevention, detection, evaluation, and management of high blood pressure in adults: a report of the American College of Cardiology/American Heart Association Task Force on Clinical Practice Guidelines. J. Am. Coll. Cardiol..

[3] Pickering T.G., Hall J.E., Appel L.J., Falkner B.E., Graves J., Hill M.N., Roccella E.J. (2005). Recommendations for blood pressure measurement in humans and experimental an imals: Part 1: Blood pressure measurement in humans: A statement for professionals from the Subcommittee of Professional and Public Education of the American Heart Association Council on High Blood Pressure Research. Circulation.

[4] Yu L., Kim B., Meng E. (2014). Chronically implanted pressure sensors: Challenges and state of the field. Sensors.

[5] Springer F., Günther R.W., Schmitz-Rode T. (2008). Aneurysm sac pressure measurement with minimally invasive implantable pressure sensors: An alternative to current surveillance regimes after EVAR?. Cardiovasc. Interv. Radiol..

[6] Van der Wel M.C., Buunk I.E., Van Weel C., Thien T.A., Bakx J.C. (2011). A novel approach to office blood pressure measurement: 30-minute office blood pressure vs daytime ambulatory blood pressure. Ann. Fam. Med..

[7] We Don’t Just Need Precision Medicine, We Need Precision Health. https://www.forbes.com/sites/valleyvoices/2016/01/06/we-dont-just-need-precision-medicine-we-need-precision-health/.

[8] Weber M.J., Yoshihara Y., Sawaby A., Charthad J., Chang T.C., Arbabian A. (2018). A Miniaturized Single-Transducer Implantable Pressure Sensor with Time-Multiplexed Ultrasonic Data and Power Links. IEEE J. Solid-State Circuits.

[9] Bartholome W.G. (1992). A revolution in understanding: how ethics has transformed health care decision making. QRB. Qual. Rev. Bull..

[10] Allen M.G. Micromachined endovascularly-implantable wireless aneurysm pressure sensors: From concept to clinic. Proceedings of the 13th International Conference on Solid-state Sensors, Actuators and Microsystems.

[11] Fonseca M.A., Allen M.G., Kroh J., White J. Flexible wireless passive pressure sensors for biomedical applications. Proceedings of the Solid-State Sensor, Actuator, and Microsystems Workshop.

[12] Fonseca M.A., English J.M., Von Arx M., Allen M.G. (2002). Wireless micromachined ceramic pressure sensor for high-temperature applications. J. Microelectromech. Syst..

[13] Fassbender H., Mokwa W., Gortz M., Trieu K., Urban U., Schmitz-Rode T., Osypka P. Fully implantable blood pressure sensor for hypertonic patients. Proceedings of the IEEE Sensors.

[14] Murphy O.H., Bahmanyar M.R., Borghi A., McLeod C.N., Navaratnarajah M., Yacoub M.H., Toumazou C. (2013). Continuous in vivo blood pressure measurements using a fully implantable wireless SAW sensor. Biomed. Microdevices.

[15] Cong P., Chaimanonart N., Ko W.H., Young D.J. (2009). A wireless and batteryless 10-bit implantable blood pressure sensing microsystem with adaptive RF powering for real-time laboratory mice monitoring. IEEE J. Solid-State Circuits.

[16] Chow E.Y., Chlebowski A.L., Chakraborty S., Chappell W.J., Irazoqui P.P. (2010). Fully wireless implantable cardiovascular pressure monitor integrated with a medical stent. IEEE Trans. Biomed. Eng..

[17] Joung Y.H. (2013). Development of implantable medical devices: From an engineering perspective. Int. Neurourol. J..

[18] Kim J.T., Kim S.I., Joung Y.H. (2013). Design and Fabrication of Implantable LC Resonant Blood Pressure Sensor. J. Korean Inst. Electr. Electron. Mater. Eng..

[19] Kim S.I., Kim E.B., So S.K., Choi J., Joung Y.H. (2016). Development of Implantable Blood Pressure Sensor Using Quartz Wafer Direct Bonding and Ultrafast Laser Cutting. J. Biome. Eng. Res..

[20] Jacobson B., Nordberg L. (1961). Endoradiosondes for pressure telemetering. IRE Trans. Bio-Med. Electron..

[21] Olsen E.R., Collins C.C., Loughborough W.F., Richards V., Adams J.E., Pinto D.W. (1967). Intracranial pressure measurement with a miniature passive implanted pressure transensor. Am. J. Surg..

[22] Yang C., Zhao C., Wold L., Kaufman K.R. (2003). Biocompatibility of a physiological pressure sensor. Biosens. Bioelectron..

[23] Lin P.-H., Huang S.-C., Chen K.-P., Li B.-R., Li Y.-K. (2019). Effective Construction of a High-Capacity Boronic Acid Layer on a Quartz Crystal Microbalance Chip for High-Density Antibody Immobilization. Sensors.

[24] Kim S., Kim J., Joung Y.-H., Choi J., Koo C. (2018). Bonding Strength of a Glass Microfluidic Device Fabricated by Femtosecond Laser Micromachining and Direct Welding. Micromachines.

[25] Suratwala T.I., Miller P.E., Bude J.D., Steele W.A., Shen N., Monticelli M.V., Wong L.L. (2011). HF-based etching processes for improving laser damage resistance of fused silica optical surfaces. J. Am. Ceram. Soc..

[26] Kim S.I., Kim J., Koo C., Joung Y., Choi J. Rapid prototyping of 2D glass microfluidic devices based on femtosecond laser assisted selective etching process. Proceedings of the SPIE 10522, Frontiers in Ultrafast Optics: Biomedical, Scientific, and Industrial Applications XVIII.

[27] Wang C., Wang Y., Tian Y., Wang C., Suga T. (2017). Room-temperature direct bonding of silicon and quartz glass wafers. Appl. Phys. Lett..

[28] Gong Y., Park J.M., Lim J. (2016). An interference-assisted thermal bonding method for the fabrication of thermoplastic microfluidic devices. Micromachines.

[29] Heptonstall A., Barton M., Cantley C., Cumming A., Cagnoli G., Hough J., Torrie C. (2010). Investigation of mechanical dissipation in CO_2_ laser-drawn fused silica fibres and welds. Classical Quantum Gravity.

[30] Sugioka K., Cheng Y. (2014). Ultrafast lasers—Reliable tools for advanced materials processing. Light Sci. Appl..

[31] Shah L., Tawney J., Richardson M., Richardson K. (2001). Femtosecond laser deep hole drilling of silicate glasses in air. Appl. Surf. Sci..

[32] Tamaki T., Watanabe W., Nishii J., Itoh K. (2005). Welding of transparent materials using femtosecond laser pulses. Jpn. J. Appl. Phys..

[33] Tamaki T., Watanabe W., Itoh K. (2006). Laser micro-welding of transparent materials by a localized heat accumulation effect using a femtosecond fiber laser at 1558 nm. Opt. Express.

[34] Miyamoto I., Cvecek K., Okamoto Y., Schmidt M. (2010). Novel fusion welding technology of glass using ultrashort pulse lasers. Phys. Procedia.

[35] Tan H., Duan J.A. (2017). Welding of glasses in optical and partial-optical contact via focal position adjustment of femtosecond-laser pulses at moderately high repetition rate. Appl. Phys. A.

[36] Gstalter M., Chabrol G., Bahouka A., Dorkenoo K.D., Rehspringer J.L., Lecler S. Optimum parameters for high-repetition rate femtosecond laser glass welding using an optical head with long focal length. Proceedings of the SPIE 10683, Fiber Lasers and Glass Photonics: Materials through Applications.

[37] Huang H., Yang L.M., Liu J. Direct welding of fused silica with femtosecond fiber laser. Proceedings of the SPIE 8244, Laser-based Micro- and Nanopackaging and Assembly VI.

[38] Miyamoto I., Cvecek K., Schmidt M. (2013). Crack-free conditions in welding of glass by ultrashort laser pulse. Opt. Express.

[39] Cvecek K., Miyamoto I., Strauss J., Wolf M., Frick T., Schmidt M. (2011). Sample preparation method for glass welding by ultrashort laser pulses yields higher seam strength. Appl. Opt..

[40] Richter S., Zimmermann F., Eberhardt R., Tunnermann A., Nolte S. (2015). Toward laser welding of glasses without optical contacting. Appl. Phys. A.

[41] Okamoto Y., Miyamoto I., Cvecek K., Okada A., Takahashi K., Schmidt M. (2013). Evaluation of Molten Zone in Micro-welding of Glass by Picosecond Pulsed Laser. J. Laser Micro/Nanoeng..

[42] Kaiser E. (2016). Laser Welding of Glass Replaces Glueing Procedure: Glass welding with a femtosecond laser brings economic advantages and new design options. Laser Tech. J..

[43] Nopper R., Niekrawietz R., Reindl L. (2010). Wireless readout of passive LC sensors. IEEE Trans. Instrum. Meas..

[44] Chen P.J., Saati S., Varma R., Humayun M.S., Tai Y.C. (2010). Wireless intraocular pressure sensing using microfabricated minimally invasive flexible-coiled LC sensor implant. J. Microelectromech. Syst..

[45] Rangsten P., Vallin O., Hermansson K., Backlund Y. (1999). Quartz-to-Quartz Direct Bonding. J. Electrochem. Soc..

[46] Cvecek K., Odato R., Dehmel S., Miyamoto I., Schmidt M. (2015). Gap bridging in joining of glass using ultra short laser pulses. Opt. Express.

[47] Chen J., Carter R.M., Thomson R.R., Hand D.P. (2015). Avoiding the requirement for pre-existing optical contact during picosecond laser glass-to-glass welding. Opt. Express.

[48] Malvindi M.A., Brunetti V., Vecchio G., Galeone A., Cingolani R., Pompa P.P. (2012). SiO_2_ nanoparticles biocompatibility and their potential for gene delivery and silencing. Nanoscale.

[49] Cvecek K., Miyamoto I., Schmidt M. (2014). Gas bubble formation in fused silica generated by ultra-short laser pulses. Opt. Express.

[50] Richter S., Döring S., Tünnermann A., Nolte S. (2010). Bonding of glass with femtosecond laser pulses at high repetition rates. Appl. Phys. A.

