# Supplementary material for: Characteristics of an Implantable Blood Pressure Sensor Packaged by Ultrafast Laser Microwelding
Source: Sensors (Basel). 2019 Apr 15;19(8):1801. doi: 10.3390/s19081801 (PMC6514925; doi:10.3390/s19081801)
Supplement: Supplementary file 1 [file sensors-19-01801-s001.pdf]

sensors

MDPI

Supplementary Materials

Characteristics of an Implantable Blood Pressure Sensor Packaged by Ultrafast Laser Microwelding

Sungil Kim [1,2], Jaesoon Park [1], Sangkyun So [1], Sanghoon Ahn [2], Jiyeon Choi [2,*], Chiwan Koo [1,*] and Yeun-Ho Joung [1,*]

1 Department of Electronics and Control Engineering, Hanbat National University, Daejeon 34158, Korea; sung1@hanbat.ac.kr (S.K.); wotns3645@naver.com (J.P.); sanggso@hanbat.ac.kr (S.S.)
2 Department of Laser and Electron Beam Application, Korea Institute of Machinery and Materials, Daejeon 34103, Korea; shahn@kimm.re.kr
* Correspondence:jchoi@kimm.re.kr (J.C.); cwankoo@hanbat.ac.kr (C.K.); yeunho@gmail.com (Y.-H. J.); Tel.: +82-42-868-7536 (J.C.); +82-42-821-1168 (C.K); +82-42-821-1166 (Y.-H. J.)

The implantable blood pressure sensor was fabricated by various MEMS processes (cleaning process, deposition, photolithography, electroplating, etching) as shown in Figure S1. This file supplements the implantable blood pressure sensor fabrication process.

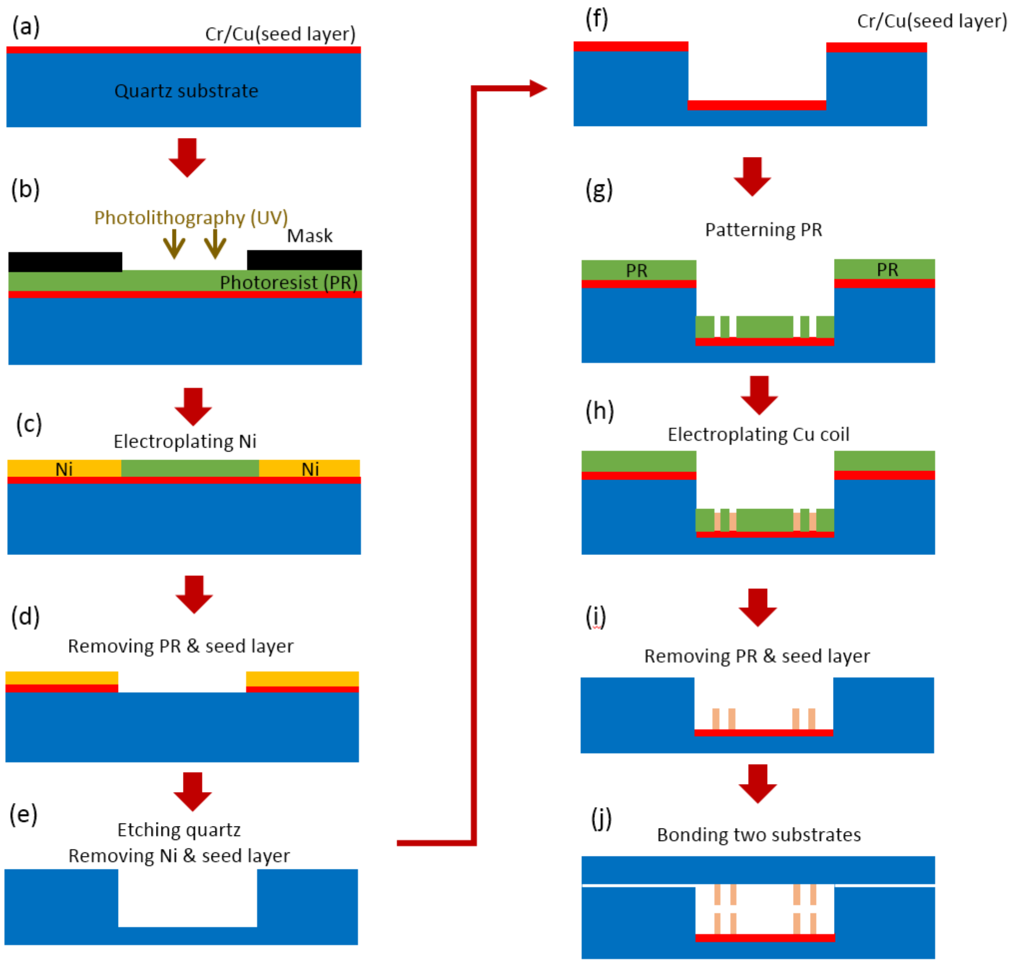

Figure S1. Implantable sensor fabrication flow.

First of all, two 4-inch quartz wafers (thickness: 500 μm) were cleaned in piranha solution for 10 minutes and rinsed with DI water. A chromium/copper (20 nm/300 nm) seed layer was deposited using a DC-sputter (Figure S1a). A negative photoresist (PR) was patterned as a mold for nickel electroplating (Figure S1b). Nickel is selected as a masking layer for quartz etching because nickel has good corrosion resistance to the etchant. Following the PR patterning, 4 μm of the nickel was electroplated (Figure S1c). After removing PR and etching the seed layer, the quartz substrate was partially etched using 40% HF (hydrofluoric acid) to create a 30 μm deep trench (Figure S1d, e). To fabricate a micro inductor on the trench bottom, a chromium/copper seed layer was deposited and a thick negative PR for forming an electroplating mold was patterned using photolithography (Figure S1f, g). After the photolithography, the inductor was electroplated (Figure S1h), and the PR and the seed layer was removed (Figure S1i). Another inductor was fabricated on a quartz substrate using the same fabrication processes (the upper substrate in Figure S1j). Before the two substrates were directly bonded (optical contact) without adhesive layer as shown in Figure S1j, DHF cleaning was conducted with 0.4 % HF to improve the substrate surface roughness and to remove the particles, which helps the good quality optical contact. After the optical contact, the sensor was welded and cut by the ultrafast laser.
